# Supplementary material for: Transcriptomic and Physiological Studies Unveil that Brassinolide Maintains the Balance of Maize’s Multiple Metabolisms under Low-Temperature Stress
Source: Int J Mol Sci. 2024 Aug 29;25(17):9396. doi: 10.3390/ijms25179396 (PMC11395154; doi:10.3390/ijms25179396)
Supplement: Supplementary file 1 [file ijms-25-09396-s001.zip › Supplementary Figures.pdf]

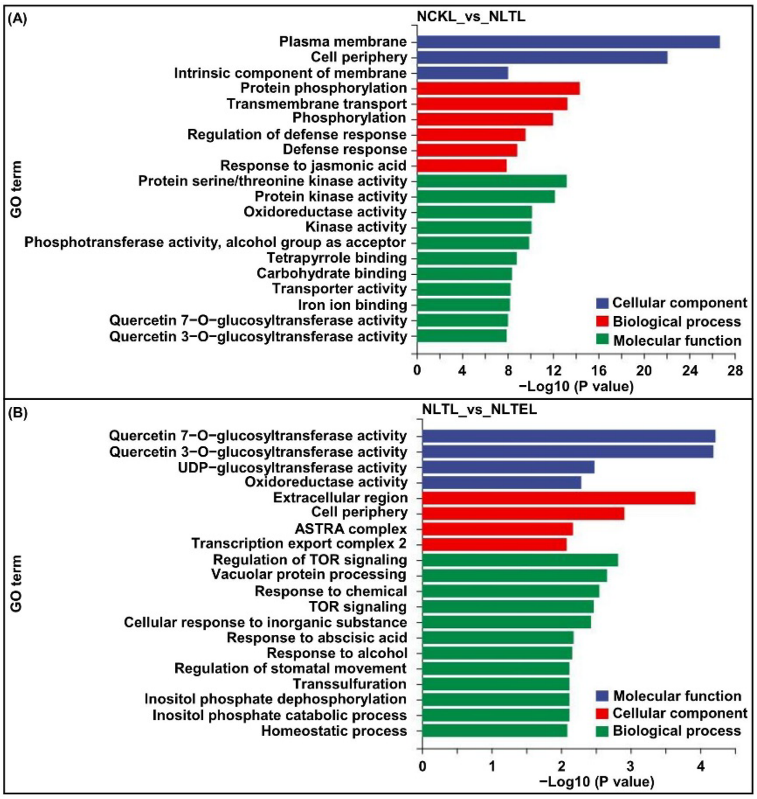

Figure S1 GO enrichment analysis of the differentially expressed genes (DEGs) detected in NCKL_vs_NLTL and NLTL_vs_NLTEL comparisons. The topmost enriched GO terms under the three main GO categories are shown. NCKL: leaves of N192 seedlings treated with 0 μM 2,4-epibrassinolide (EBR) application at 25°C normal temperature (CK); NLTL: leaves of N192 seedlings treated with 0 μM EBR application at 15°C low-temperature (LT); NLTEL: leaves of N192 seedlings treated with 2 μM EBR application at 15°C normal temperature (LTE).

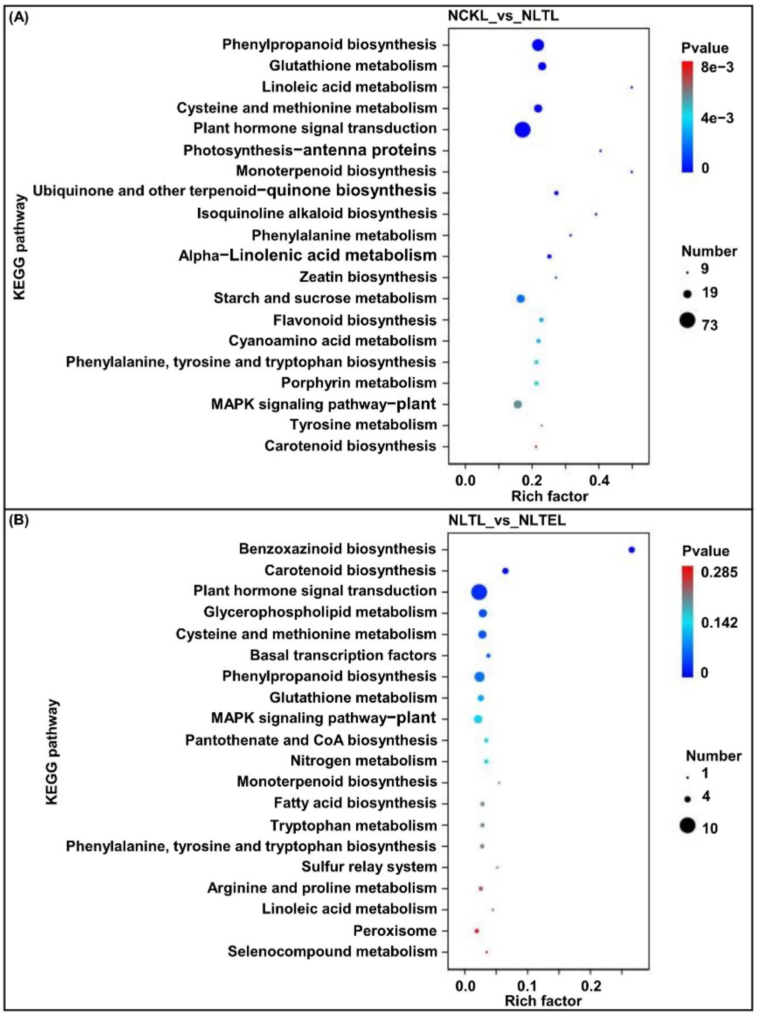

Figure S2 The top20 KEGG pathway enrichment analysis of the differentially expressed genes (DEGs) detected in NCKL_vs_NLTL and NLTL_vs_NLTEL comparisons. Rich factor is the ratio of DEGs to the total number of genes in each pathway. NCKL: leaves of N192 seedlings treated with 0 μM 2,4-epibrassinolide (EBR) application at 25°C normal temperature (CK); NLTL: leaves of N192 seedlings treated with 0 μM EBR application at 15°C low-temperature (LT); NLTEL: leaves of N192 seedlings treated with 2 μM EBR application at 15°C normal temperature (LTE).

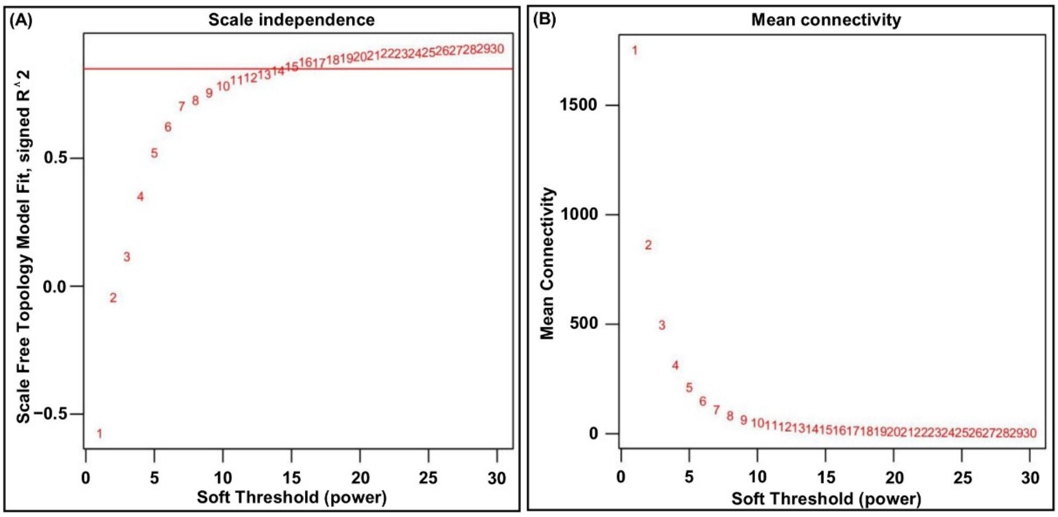

Figure S3 Soft threshold determination of gene co-expression network. (**A**) The abscissae is the soft threshold (β) and the ordinate is the scale-free network model index. (**B**) The abscissae is the soft threshold (β) and the ordinate is the mean connectivity.

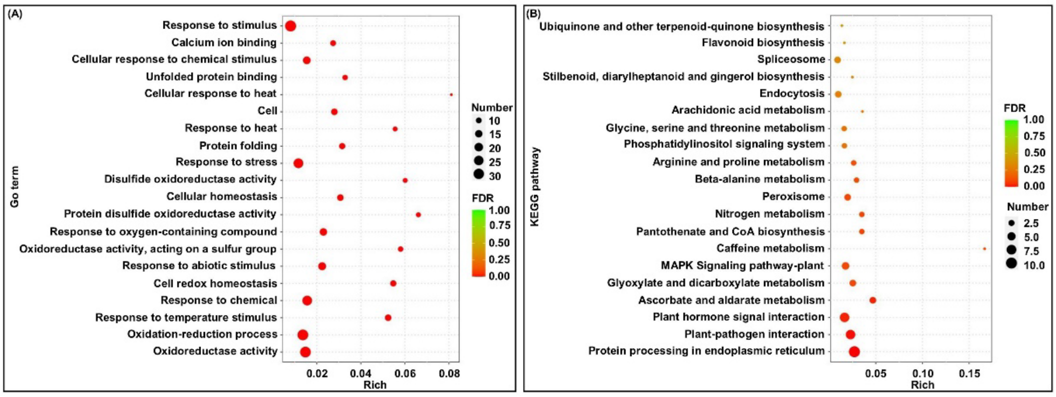

Figure S4 The function enrichment annotations of identified hub genes in four modules (blue, brown, salmon, and red). (**A**) GO enrichment analysis of these hub genes. (**B**) The top20 KEGG pathway enrichment analysis of these hub genes.
